# Supplementary material for: The Modulatory Effects of DMF on Microglia in Aged Mice Are Sex-Specific
Source: Cells. 2022 Feb 18;11(4):729. doi: 10.3390/cells11040729 (PMC8870377; doi:10.3390/cells11040729)
Supplement: Supplementary file 1 [file cells-11-00729-s001.zip › cells-1574525-supplementary.pdf]

cells

MDPI

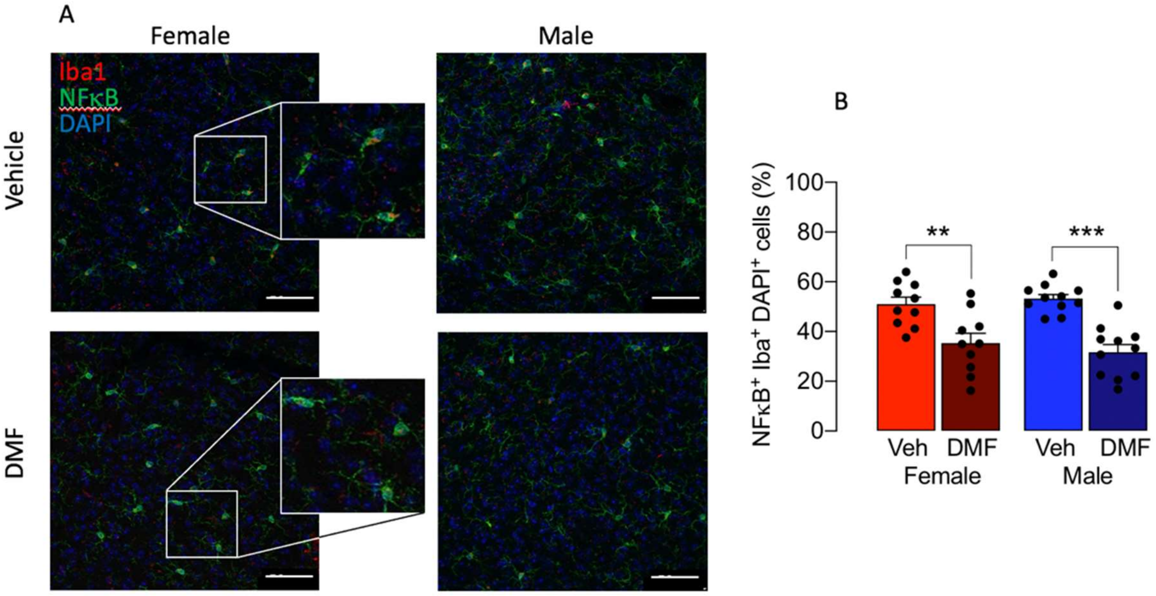

Supplementary Figure S1. DMF decreases translocation of NFkB to the nucleus in Iba1+ microglia in cortical tissue. **A**. p65 NFκB expression in DAPI-stained nuclei was assessed in Iba1+ microglia in cortical sections prepared from DMF- and vehicle-treated control male and female mice. The sample immunographs indicate that DMF decreased NFκB staining in sections from male and female mice, and this is also shown in the higher magnification insets from sections of female mice. Scale bars = 50 μm. **B**. Analysis of the mean data revealed that there was a significant main effect of treatment in NFκB staining ($p < 0.001$; 2-way ANOVA) and post hoc analysis indicated that p65 NFκB+ DAPI+ staining in Iba1+ cells was significantly decreased in sections from DMF-treated male and female mice compared with controls (** $p < 0.01$; *** $p < 0.001$) Data are expressed as a percentage of the total number of Iba1+ microglia and are presented as means ± SEM (n = 10 or 11).

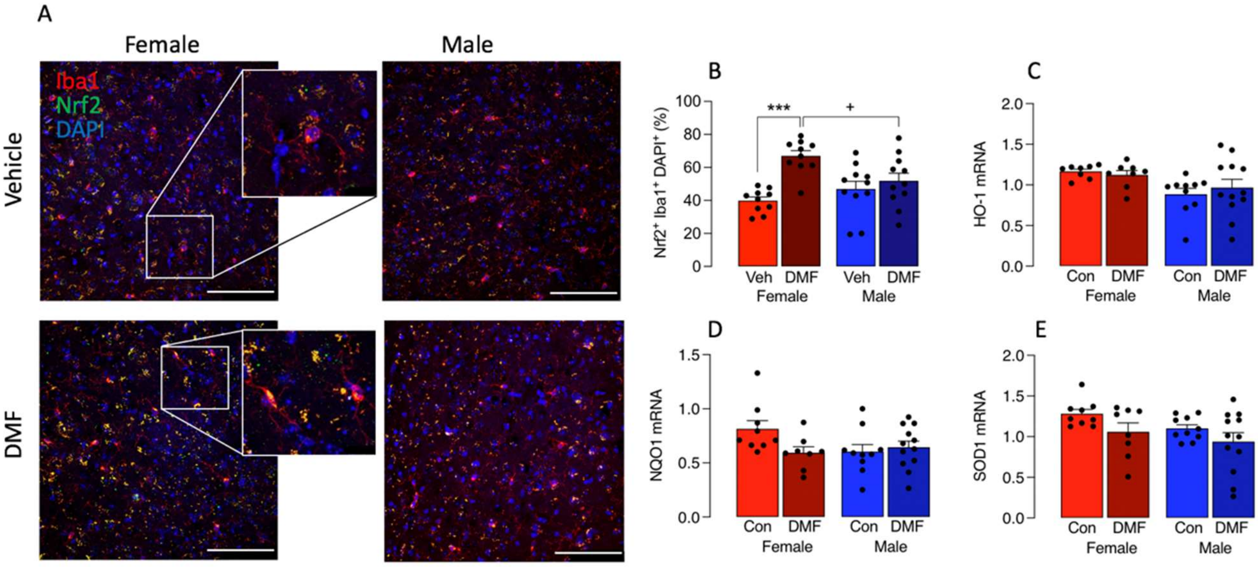

Supplementary Figure S2. DMF increases translocation of Nrf2 to the nucleus in Iba1+ microglia in cortical tissue. **A**. Nrf2 expression in DAPI-stained nuclei was assessed in Iba1+ microglia in cortical

sections prepared from DMF- and vehicle-treated control male and female mice. The sample immunographs indicate that DMF increased Nrf2 staining particularly in sections from female mice and this is also shown in the higher magnification insets. Scale bars = 50 μm **B**. Analysis of the mean data revealed that there was a significant treatment x sex interaction ($p < 0.01$; 2-way ANOVA) and post hoc analysis indicated that DMF significantly increased $Nrf2^+$ $DAPI^+$ staining in $Iba1^+$ cells in sections from female mice (*** $p < 0.001$; D), and that there was a significant difference in staining in sections from DMF-treated females compared with males ($^+ p < 0.05$). Data are expressed as a percentage of the total number of Iba1+ microglia and are presented as means ± SEM (n = 10 or 11). **C–E**. A significant main effect of sex was observed in HO-1 mRNA ($p < 0.05$; C) and significant main effect of DMF was observed in SOD mRNA ($p < 0.05$; E). No sex- or treatment-related effects were observed in NQO1 mRNA.
